# Supplementary material for: Single-Nucleus Transcriptome Sequencing Unravels Physiological Differences in Holstein Cows Under Different Physiological States
Source: Genes (Basel). 2025 Aug 3;16(8):931. doi: 10.3390/genes16080931 (PMC12385990; doi:10.3390/genes16080931)
Supplement: Supplementary file 1 [file genes-16-00931-s001.zip › Supplementary Figure S1.pdf]

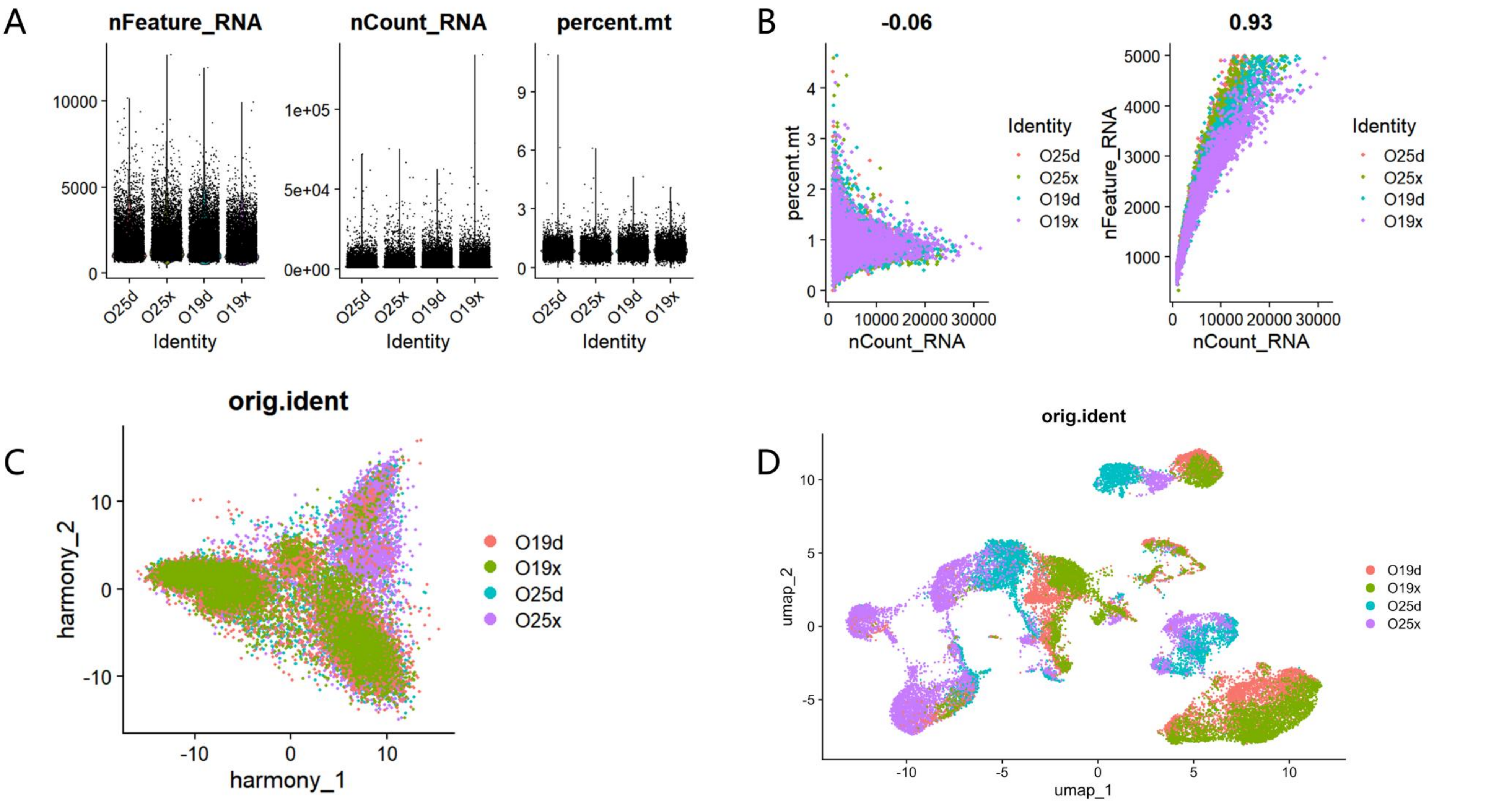


Supplementary Figure S1. Quality control of bovine ovarian single cell data. ( A ) The scatter plot shows the number of reads ( nCount _ RNA ), the number of genes ( nFeature _ RNA ) and the proportion of mitochondrial genes ( percent.mt ) in each ovarian cell of O25d, O25x, O19d and O19x. ( B ) The scatter plot shows the relationship between the number of reads, the number of genes and the proportion of mitochondrial genes.( C ) The corrected ( PCA diagram ) Harmony diagram shows the distribution of bovine ovarian tissue sample cells, ( D ) UMAP diagram shows the distribution of bovine ovarian tissue sample cells.
