# Supplementary material for: Single-Nucleus Transcriptome Sequencing Unravels Physiological Differences in Holstein Cows Under Different Physiological States
Source: Genes (Basel). 2025 Aug 3;16(8):931. doi: 10.3390/genes16080931 (PMC12385990; doi:10.3390/genes16080931)
Supplement: Supplementary file 1 [file genes-16-00931-s001.zip › Supplementary Figure S2.pdf]

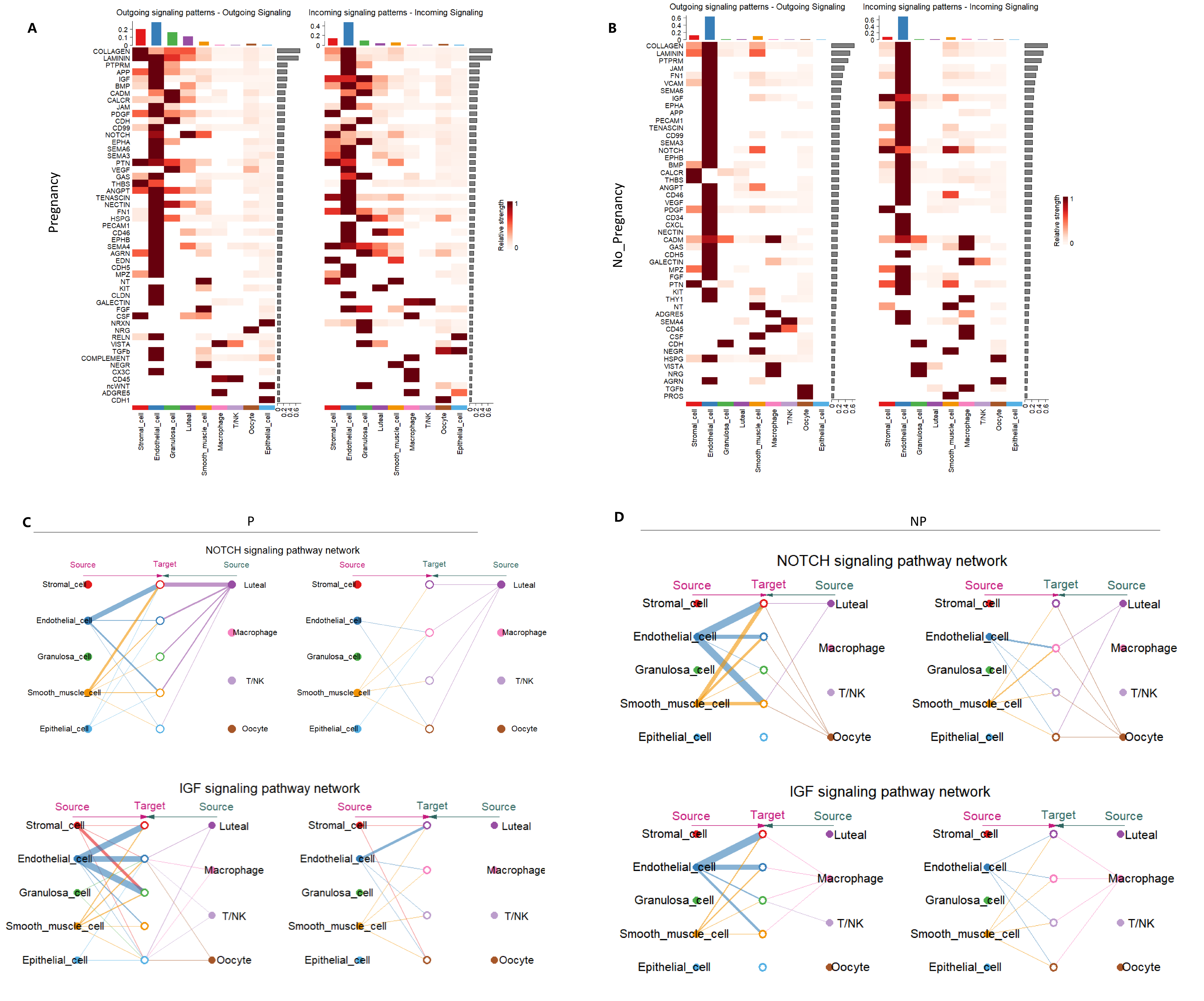

Supplementary Figure S2. (A) Heat map of all signaling pathways involved in pregnancy. (B) Heat map of all signaling pathways involved in non-pregnancy. (C) Hierarchical plot shows the intercellular communication networks of the IGF and NOTCH signaling pathways in the ovaries of Holstein cows during pregnancy. The size of the circles represents the number of cells, and the width of the edges represents the communication probability. (D) Hierarchical plot shows the intercellular communication networks of the IGF and NOTCH signaling pathways in the ovaries of Holstein cows during non-pregnancy. The size of the circles represents the number of cells, and the width of the edges represents the communication probability.
