# Supplementary figures and images for: Single-Nucleus Transcriptome Sequencing Unravels Physiological Differences in Holstein Cows Under Different Physiological States
Source: Genes (Basel). 2025 Aug 3;16(8):931. doi: 10.3390/genes16080931 (PMC12385990; doi:10.3390/genes16080931)

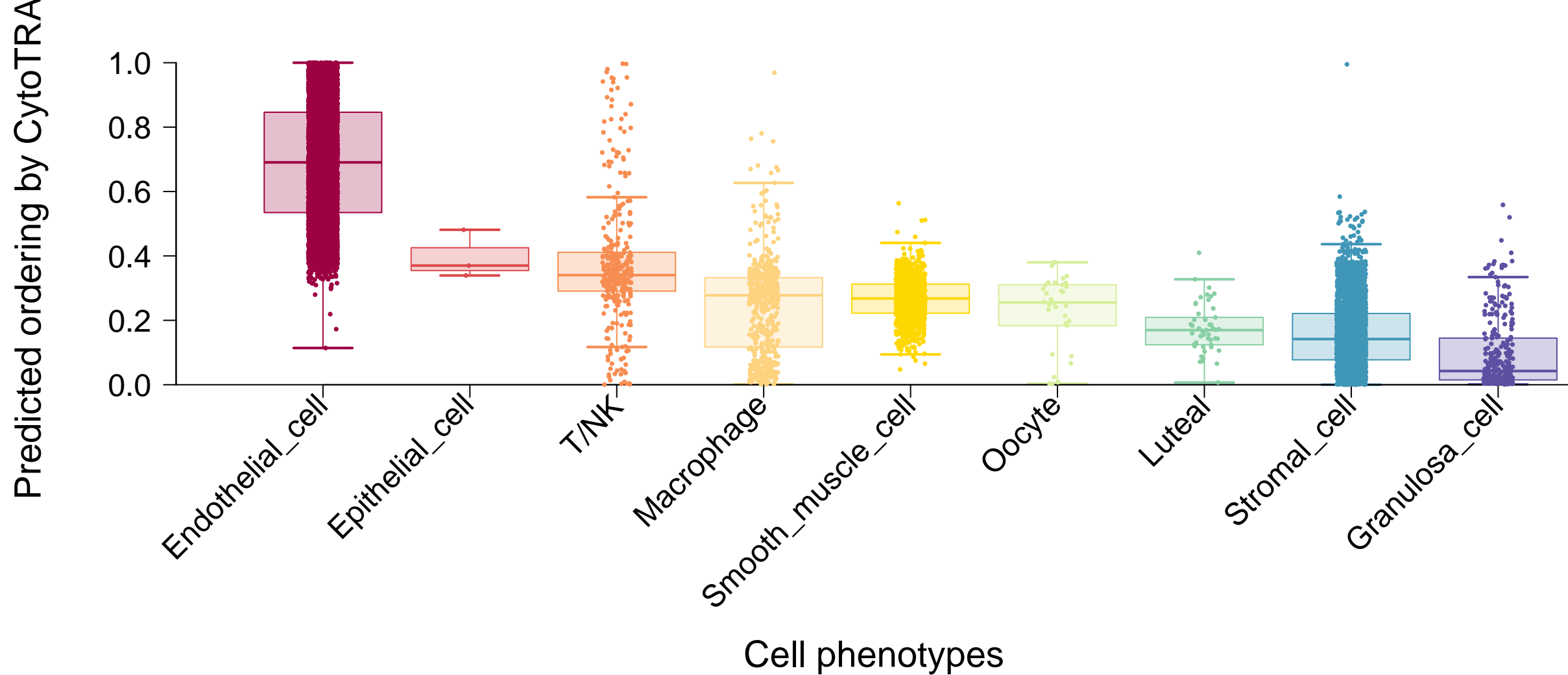

Supplement: Supplementary file 1 [file genes-16-00931-s001.zip › Supplementary Figure S3.pdf]

Predicted ordering by CytoTRA

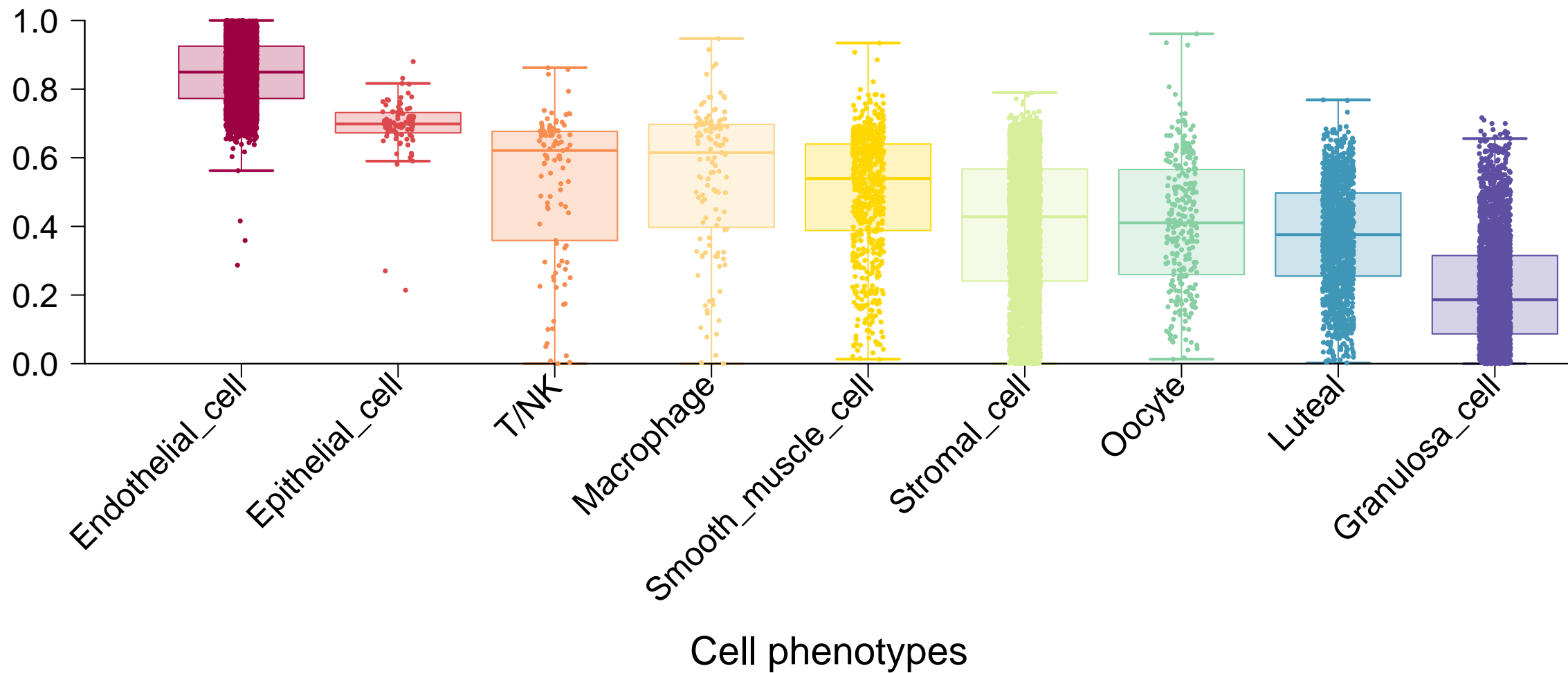

Supplement: Supplementary file 1 [file genes-16-00931-s001.zip › Supplementary Figure S4.pdf]
